# Analytical Value of Cell-Free DNA Based on *Alu* in Psychiatric Disorders

**DOI:** 10.3389/fpsyt.2019.00992

**Published:** 2020-01-21

**Authors:** Jing Qi, Ling-Yun Chen, Xian-Juan Shen, Shao-Qing Ju

**Affiliations:** ^1^ Research Center of Clinical Medicine, Affiliated Hospital of Nantong University, Nantong, China; ^2^ Center of Laboratory Medicine, Nantong Mental Health Center, Nantong, China; ^3^ Center of Laboratory Medicine, Affiliated Hospital of Nantong University, Nantong, China

**Keywords:** biomarker, *Alu*, psychiatric disorders, interleukin-1β, interleukin-18

## Abstract

Psychiatric disorders impose a huge burden on individuals, families, and society. The *Alu* repeat sequence is a member of the short interspersed nuclear element (SINE) family of mammalian genomes, however, its expression pattern and role in psychiatric disorders is unclear. The current paper aimed at determining the concentrations of *Alu* in patients with schizophrenia (SZ), major depressive disorder (MDD), and alcohol-induced psychotic disorder (AIPD), and to further define the role and value of *Alu* as a potential biomarker in psychiatric disorders. In this work, we found that the concentration of *Alu* was considerably incremented in patients with SZ, and a significant difference existed between patients diagnosed with SZ and MDD or AIPD. ROC analysis also indicated that *Alu* was effective in the complementary diagnosis of SZ, and differentially diagnosed between SZ patients and patients with MDD or AIPD. In addition, we found a positive relationship between the *Alu* concentrations and interleukin-1β (IL-1β) in patients with SZ, MDD, and AIPD, and between the concentrations of *Alu* and interleukin-18 (IL-18) in patients with SZ. Overall, the present work indicates that *Alu* might be an innovative biomarker for diagnosing psychiatric disorders, and provides the basis for hypotheses about the pathophysiology of psychiatric disorders.

## Introduction

The development and progression of psychiatric disorders, whose clinical signs include disorders in emotion, cognition, and behavior, are related to genetic and environmental factors. The total prevalence of psychiatric disorders is 17% in China (1) and the prevalence of psychiatric disorders is increasing in each year (2). Huge burdens are imposed by psychiatric disorders on families, individuals, and society. The psychiatric disorders burden is about 13% in China, ranking third in the world after cardiovascular disease and cancer. Schizophrenia (SZ), major depressive disorder (MDD), and alcohol-induced psychotic disorder (AIPD) are the main types of psychiatric disorders in Nantong, Jiangsu, China. The underlying causes of psychiatric disorders are unidentified in 90% of the cases. Diagnosis mainly depends on clinical symptoms, prognosis, and course of the disease, however, they are not specific for psychiatric disorders. Some characteristics are applied to SZ, MDD, and AIPD. Difficulties exist in the diagnosis and treatment of psychiatric disorders, which are based merely on clinical indications. Serological biomarkers are precise, objective, and easy to apply, with wide applications in various diseases like inflammation, tumors, and cardiovascular diseases. Therefore, it is worthwhile to seek innovative molecular markers for psychiatric disorders (3–6).

The pathophysiology of psychiatric disorders is still mysterious. Presently, neuronal apoptosis is regarded as one of the mechanisms of psychiatric disorders, in general. The apoptosis levels incremented in patients suffering from psychiatric disorders and the succeeding neuronal, dendritic, and synaptic losses are caused by an incremented or imbalanced apoptotic mechanism (7–9). Glutamate excitotoxicity, oxidative stress, and decreased neurotrophic support that are linked to psychiatric disorders, also directly or indirectly related to the apoptosis (10–12). There are several studies reporting some genes associated with apoptosis, which were changed in patients with SZ, like *Bcl-2* (13) and down-expressed GSK3 levels (14–16) and increased *Bax*/*Bcl-2* ratios, caspase-3 activity (17), and the p53 expression level (18–21). The pathogenesis of neuropsychiatric diseases such as depression and anxiety is also associated with apoptosis (22–25).

The *Alu* repeat sequence is a member of the short interspersed nuclear element (SINE) family of mammalian genomes, and it is expressed exclusively by primates. It can be released from apoptotic cells. Several copies of unedited *Alu* repeats were found by Hardy et al., contained in exosome-like nanovesicles (ApoExos), which were released by apoptotic endothelial cells (26). *Alu* has been utilized as a biomarker in many kinds of diseases including cancer (27, 28). The level of *Alu* is increased significantly in patients with cancer, in comparison with healthy individuals, and after treatment, it is decreased considerably (29). High levels of *Alu* are related to a poor prognosis of patients. The *Alu* concentration is a promising molecular marker to assess cancer progression (30). Nevertheless, the concentrations and role of *Alu* in SZ, MDD, and AIPD are not clear.

The present work aimed at determining the concentrations of *Alu* in patients with SZ, MDD, and AIPD, and further exploring the value and role of *Alu* as a potential biomarker in psychiatric disorders. Based on our result, the concentration of *Alu* was considerably incremented in patients with SZ, and we found a considerable difference between SZ patients and MDD or AIPD patients. Moreover, ROC analysis also indicated that *Alu* was helpful in the complementary diagnosis of SZ, and differential identification between patients with SZ and patients with MDD or AIPD. Additionally, we found a positive relationship between interleukin-1β (IL-1β) and the concentrations of *Alu* in patients with SZ, MDD, and AIPD, and between the concentrations of *Alu* and interleukin-18 (IL-18) in patients with SZ. In sum, this work offers an innovative biomarker in diagnosing the psychiatric disorders and a promising hypothesis of the pathophysiology of psychiatric disorders.

## Materials and Methods

### Human Serum Samples

The samples were taken from 164 SZ patients, 48 MDD patients, and 29 AIPD patients from the Nantong Fourth Peoples Hospital (Nantong, China) between Dec 2017 to Aug 2018. All patients with psychiatric disorders confirmed in terms of the criteria of Diagnostic and Statistical Manual of Mental Disorders, Four Edition. One hundred healthy individuals were gathered from the Physical Examination Center of the Affiliated Hospital of Nantong University (Nantong, China) who had passed the physical test. **Table 1** shows the features of the human samples. Before collecting the sample, informed consent was obtained. All samples were anonymous and the study was permitted by the Human Research Ethics Committee of the Affiliated Hospital of Nantong University. The disposable vacuum blood collection tubes and blood collection needles were used for blood specimen collection. The procedure is strictly in accordance with the Guidelines for the Collection of Adult Venous Blood Specimens (GB/T 1.1-2009). The specimen tube was erected and left to avoid oscillation, falling, and direct sunlight after blood collecting. During operation, protective clothing, safety goggles, or face shields, as well as clean disposable gloves and masks, were worn. Moreover, clean disposable tips and centrifuge tubes were used. The gloves were removed and the hands were washed or disinfected immediately after the operation. The clothes, gloves, and other items contaminated by blood were put into the medical waste garbage bag. The sharps were placed into a special anti-seepage and stab-resistant disposable sharp box. Both the garbage bags and the sharp boxes were sealed and treated by centralized medical waste treatment of our hospital. The contaminated tabletops were cleaned with disinfectant such as hypochlorous acid.

**Table 1:** Demographic details of participant.

	Healthy individuals	SZ patients	MDD patients	AIPD patients
Total Number [N]	100	164	48	29
Gender [M:F]	47:53	85:79	16:32	29:0
Mean of Age (years)	34.8	26.7	45.3	50.0

SZ, schizophrenia; MDD, major depressive disorder; AIPD, alcohol-induced psychotic disorder.

### Serum Collection and Cell-Free DNA Isolation

Blood samples (4–5 ml) directly were gathered into serum separator tubes (Vacuette, Kresmunster, Austria). The complete blood was centrifuged at 3000×g for 10 min, and the serum was stored at −80°C till being used. Using the TIAN LONG DNA Kit (Suzhou, China) based on the protocol of the manufacturer, cell-free DNA was extracted from 0.2 ml of serum.

### Quantitation of *Alu*

The concentration of *Alu* was quantitated by real-time PCR (RT-PCR) established by Hao TB et al. in our study team (31). RT-PCR was performed in triplicate with FastStart Universal SYBR Green Master Mix kit (Roche, Germany) by 7500 RT-PCR System (ABI, Abilene, TX, USA). A standard curve (from 0.222 to 22 200 ng/ml) of human genomic DNA (Promega, Madison, WI, USA) was used to quantify the absolute level of *Alu*. The sequence of *Alu-*115 forward primer was 5'-CCTGAGGTCAGGAGTTCGAG-3' and the reverse primer was 5'-CCCGAGTAGCTGGGATTACA-3'. A 20 µl PCR mixture contained 5 µl DNA template, 0.5 µl of the forward and reverse primer (10 μM), 10 µl SYBR Green Master Mix, and 4 µl ddH_2_O at 95°C for 10 min, followed by 35 cycles of denaturation at 95°C for 15 s, and annealing at 64°C for 1 min.

### Quantizing the IL-18 and IL-1β

The IL-18 and IL-1β expression levels were found by the Human IL-18 ELISA kit (Fcmacs Technology, China) and Human IL-1β ELISA kit (Fcmacs Technology, China) based on the manufacturer protocols, respectively.

### Statistical Analysis

Using GraphPad Prism v5.0 software, statistical analysis was conducted. The concentrations of *Alu*, IL-18, and IL-1β are provided as the median with the 25th and the 75th percentile values. The nonparametric quantitative information was compared in the form of inter-group through the Mann Whitney test. The nonparametric quantitative data of more than two groups were compared using the Kruskal-Wallis test. *Via* the Spearman test, the correlation was examined. To investigate the diagnostic accurateness of each parameter, receiver operating characteristic curves (ROC) were made, and the specificity and sensitivity of the optimum cut-off point were determined as values maximizing the ROC curve (AUC) area. The confidence interval of ROC was determined as 95%. All statistical examinations were two-sided, and the statistical significance implied a P-value less than 0.05.

## Results

### The Concentrations of *Alu* in Patients with SZ, MDD, AIPD, and Healthy Individuals

**Figure 1:**
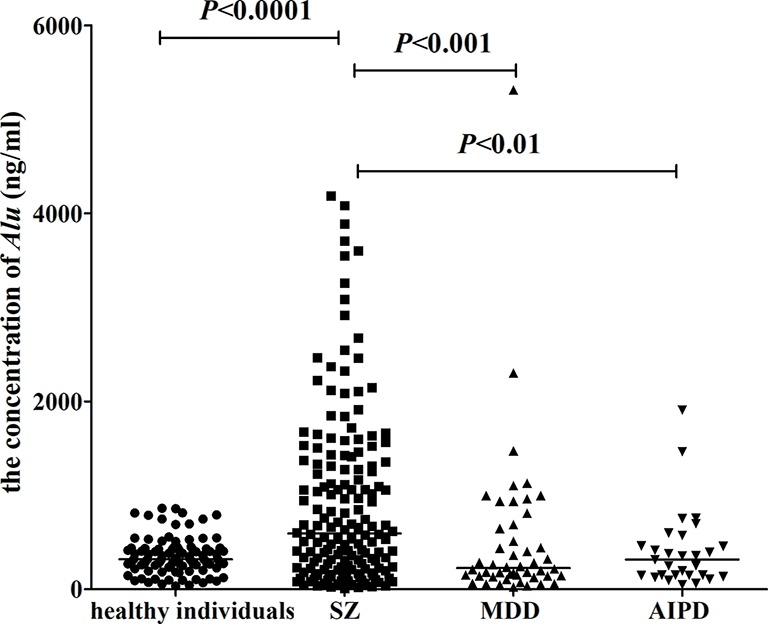
The concentrations of *Alu* in healthy individuals, patients with SZ, MDD, and AIPD. Mann Whitney test was used in this figure. The concentrations of *Alu* were measured in 164 SZ patients, 48 MDD patients, 29 AIPD patients, and 100 healthy individuals. The results for the concentrations of *Alu* are presented as the median with the 25^th^ and the 75^th^ percentile values. Horizontal lines indicate the median for each group. SZ, schizophrenia; MDD, major depressive disorder; AIPD, alcohol-induced psychotic disorder.

In this work, 164 patients with SZ, 48 patients with MDD, and 29 patients with AIPD were included from the Nantong Fourth Peoples Hospital (Nantong, China). One hundred healthy individuals were gathered from the Physical Examination Center of the Affiliated Hospital of Nantong University (Nantong, China). **Table 1** represents the demographic details of the participants. The average concentrations of *Alu* in patients suffering from SZ, MDD, AIPD, and healthy people were 593.5 ng/ml (interquartile range 226.8–1,337.1 ng/ml), 225.2 ng/ml (interquartile range 139.8–655.3 ng/ml), 315.3 ng/ml (interquartile range 148.5–462.9 ng/ml), and 318.3 ng/ml (interquartile range 253.5–427.4 ng/ml), respectively. The significant difference was found between patients with SZ and healthy individuals (*P* < 0.0001), and also between SZ patients and patients with MDD (*P* < 0.001) or patients with AIPD (*P* < 0.01). However, no statistical difference was found among patients with MDD, AIPD, and healthy people (*P* > 0.05) (**Figure 1**).

### The Utility of *Alu* in Diagnosis and Differential Diagnosis

**Figure 2:**
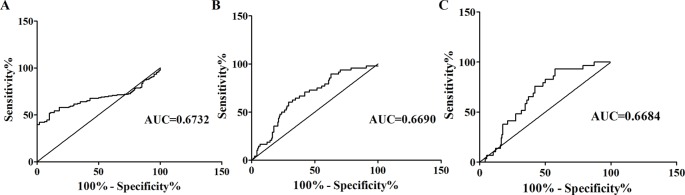
ROC curves analysis of *Alu*. **(A)** ROC curves analysis of *Alu* between SZ patients and healthy individuals. AUC was 0.6732 in separating patients with SZ from healthy individuals (95% confidence interval, 0.6091–0.7372). **(B)** ROC curves analysis of *Alu* between SZ patients and MDD patients. AUC was 0.6690 in separating patients with SZ from patients with MDD (95% confidence interval, 0.5860–0.7519). **(C)** ROC curves analysis of *Alu* between SZ patients and AIPD patients. AUC was 0.6684 in separating patients with SZ from patients with AIPD (95% confidence interval, 0.5783–0.7586). ROC, receiver operating characteristic; AUC, the area under the ROC curve; SZ, schizophrenia; MDD, major depressive disorder; AIPD, alcohol-induced psychotic disorder.

In patients with SZ, the concentrations of *Alu* were considerably increased. Whether or not *Alu* can contribute to the complementary diagnosing SZ and differential diagnoses between patients with SZ and MDD or AIPD was still undetermined. The diagnostic efficiency of *Alu* was assessed using Receiver Operating Characteristics (ROC) analysis. The (AUC) analysis curve area obtained a value of 0.6732 (95% CI: 0.6091–0.7372) between SZ patients and healthy people (**Figure 2A**). The sensitivity was 51.83% and the specificity was 90% when the cut-off value was 567.1 ng/ml. The AUC was 0.6690 in sorting out the SZ patients from MDD patients with a 60.42% sensitivity and a 70.73% specificity at the cut-off value (288 ng/ml) (**Figure 2B**), and 0.6684 in sorting out SZ patients from AIPD patients with a 75.86% sensitivity and a 57.93% specificity at the considered cut-off value (469.7 ng/ml) (**Figure 2C**).

### Analysis Between *Alu* and Clinical Characteristics of Patients with SZ, MDD, and AIPD

**Tables 2**–**4** represent the concentrations of *Alu* in various subdivisions of patients suffering from SZ, MDD, and AIPD based on gender, current age, psychosis onset age, current treatment, duration of psychosis and pharmacological treatment, and family history. No statistically considerable difference was found in *Alu* in these various classes based on gender (*P* > 0.05), current age (*P* > 0.05), psychosis onset age (*P* > 0.05), duration of pharmacological treatment (*P* > 0.05), duration of psychosis (*P* > 0.05), current treatment (*P* > 0.05), and family history (*P* > 0.05).

**Table 2:** Clinical features of patients of SZ (n = 164) according to the specific diagnostic categories.

	N	*Alu* Median (interquartile range) (ng/ml)	*P-value*
Total	164	593.5 (226.8–1337.1)	
Gender			0.4239
Male	85 (51.8%)	551.9 (229.7–1,167.5)	
Female	79 (48.2%)	669.4 (216.9–1,544.1)	
Psychosis onset age (y)			0.8873
Up to 16	47 (28.7%)	1,059.9 (534.5–1,653.9)	
17 to 24	73 (44.5%)	1,093.9 (424.1–1,584.6)	
25 to 34	36 (22.0%)	1,185.7 (545.4–1,841.3)	
35 or more	8 (4.9%)	695.9 (299.4–1,567.8)	
Current age (y)			0.0518
13 to 24	83 (50.6%)	1,061.6 (412.1–1,657.1)	
25 to 34	53 (32.3%)	1,040.4 (497.2–1,579.0)	
35 or more	28 (17.1%)	407.6 (238.2–872.0)	
Duration of psychosis (in weeks)			0.1648
Up to 12	38 (23.2%)	1,410.3 (585.7–2,014.1)	
13 to 52	22 (13.4%)	476.4 (242.8–1,314.0)	
53 or more	104 (63.4%)	1,061.4 (534.2–1,548.2)	
Pharmacological treatment (in weeks)			0.4203
Up to 4	72 (43.9%)	852.7 (476.4–1,431.4)	
5 or more	92 (56.1%)	1,090.8 (530.9–1,660.4)	
Current treatment			0.3964
AP	119 (72.6%)	1,061.8 (437.2–1,589.0)	
AP+AD	23 (14.0%)	744.9 (484.5–2,055.4)	
AP+MS	22 (13.4%)	1,227.7 (841.2–1,942.4)	
Family history			0.3730
Yes	20 (12.2%)	1,015.1 (412.1–1,412.3)	
No	144 (87.8%)	1,087.8 (484.5–1,657.1)	

SZ, schizophrenia; AP, anti-psychotics; AD, antidepressants; MS, mood stabilizers. P < 0.05 was considered statistically significant.

**Table 3:** Clinical features of patients of MDD (n = 48) according to the specific diagnostic categories.

	N	*Alu* Median (interquartile range) (ng/ml)	*P-value*
Total	48	225.2 (139.8–655.3)	
Gender			0.5548
Male	16 (33.3%)	205.8 (60.9–935.7)	
Female	32 (66.7%)	225.2 (157.3–543.6)	
Psychosis onset age (y)			0.1088
Up to 16	6 (12.5%)	966.6 (773.1–2,075.7)	
17 to 34	9 (18.75%)	340.2 (206.0–482.7)	
35 or more	33 (68.75%)	229.1 (164.1–594.3)	
Current age (y)			0.0931
13 to 34	12 (25.0%)	936.5 (279.2–996.8)	
35 or more	36 (75.0%)	251.6 (164.1–611.2)	
Duration of psychosis (in weeks)			0.9195
Up to 12	7 (14.6%)	299.6 (107.7–782.4)	
13 to 52	18 (37.5%)	242.5 (147.3–665.5)	
53 or more	23 (39.6%)	209.3 (155.0–421.8)	
Pharmacological treatment (in weeks)			0.7453
Up to 4	37 (77.1%)	251.6 (137.9–543.6)	
5 or more	11 (22.9%)	198.7 (157.0–707.3)	
Current treatment			0.4513
AD	22 (45.8%)	181.3 (95.8–665.5)	
AP+AD	22 (45.8%)	242.5 (168.6–418.4)	
AP+AD+MS	4 (8.3%)	345.7 (180.4–1,710.5)	
Family history			0.3321
Yes	4 (8.3%)	141.7 (130.5–269.0)	
No	44 (91.7%)	238.6 (147.8–716.9)	

MDD, major depressive disorder; AP, anti-psychotics; AD, antidepressants; MS, mood stabilizers.

P < 0.05 was considered statistically significant.

**Table 4:** Clinical features of patients of AIPD (n = 29) according to the specific diagnostic categories.

	N	*Alu* Median (interquartile range) (ng/ml)	*P-value*
Total	29	315.3 (148.5–462.9)	
Gender			–
Male	29 (100%)	315.3 (148.5–462.9)	
Female	0 (0%)	0	
Psychosis onset age (y)			0.8399
Up to 35	5 (17.2%)	151.8 (135.8–462.9)	
36 or more	24 (82.8%)	336.2 (150–486.6)	
Current age (y)			0.9714
30 to 35	3 (10.3%)	151.8 (143.8–454.9)	
36 or more	26 (89.7%)	336.2 (149.08–461.7)	
Duration of psychosis (in weeks)			0.4785
Up to 52	9 (31.0%)	151.8 (143.4–322.5)	
53 or more	20 (69.0%)	283.2 (148.3–461.7)	
Pharmacological treatment (in weeks)			0.7513
Up to 4	19 (65.5%)	250.7 (147.5–458.0)	
5 or more	10 (34.5%)	251.0 (139.4–347.7)	
Current treatment			0.2175
AP+AD	25 (86.2%)	251.0 (148.5–416.5)	
AP+AD+MS	4 (13.8%)	531.4 (379.2–926.7)	
Family history			–
Yes	0 (0%)	–	
No	29 (100%)	315.3 (148.5–462.9)	

AIPD, alcohol-induced psychotic disorder; AP, anti-psychotics; AD, antidepressants; MS, mood stabilizers.

P < 0.05 was considered statistically significant.

In the present work, the correlations between *Alu* and Clinical Global Impression (CGI) score, abandon score, and attack score of patients with SZ were also examined and no significant difference was found (**Table 5**).

**Table 5:** Correlation analysis between *Alu* and scores in patients with SZ.

	CGI score		Abandon score		Attack score	
	r	*P*	r	*P*	r	*P*
*Alu* (ng/ml)	−0.008242 0.9433		−0.08017 0.4883		−0.1531 0.1838	

SZ, schizophrenia; CGI, Clinical Global Impression.

P < 0.05 was considered statistically significant.

### Analysis Between *Alu* and IL-1β, IL-18 in Patients with SZ, MDD, and AIPD

It was reported that inflammatory cytokines IL-1β (32, 33) and IL-18 (34, 35) played a role in the development of psychiatric disorders. Some studies indicated that *Alu* RNA was capable of up-regulating IL-18 and IL-1β expression and secretion, and the apoptosis was caused subsequently (36, 37). However, enhancement in the expression of IL-1β and IL-18 in psychiatric disorders by *Alu* is still unclear. To clarify this matter, a correlation analysis was carried out in this work, between *Alu* and IL-1β or IL-18 in patients with SZ, MDD, and AIPD. We found that a positive correlation existed between the *Alu* and IL-1β concentrations in patients with SZ (*P* < 0.05), MDD (*P* < 0.05), and AIPD (*P* < 0.05), and between the concentration of *Alu* and IL-18 in patients with SZ (*P* < 0.05). However, there was no correlation between the concentrations of *Alu* and IL-18 in patients with MDD and AIPD (*P* > 0.05) (**Table 6**).

**Table 6:** Correlation analysis between *Alu* and IL-1β, IL-18 in patients with SZ, MDD, and AIPD.

	SZ *Alu*(ng/ml)		MDD *Alu*(ng/ml)		AIPD *Alu*(ng/ml)	
	r	*P*	r	*P*	r	*P*
IL-1β (pg/ml)	0.3699	**0.0242**	0.4408	**0.0242**	0.8110	**0.0004**
IL-18 (pg/ml)	0.3298	**0.0404**	0.5385	0.0576	−0.5105	0.0899

SZ, schizophrenia; MDD, major depressive disorder; AIPD, alcohol-induced psychotic disorder; IL-1β, interleukin-1β; IL-18, interleukin-18.

P < 0.05 was considered statistically significant.

Based on these findings, our study indicates that the concentration of *Alu* might be useful biomarker for the diagnosis of psychiatric disorders. Further, the present results may suggest novel hypotheses for the pathogenesis of psychiatric disorders.

## Discussion

Recent studies indicated numerous molecular changes in psychiatric disorders, and psychiatric disorders development and progression have a role in the abnormal expression pattern (38–40). In this study, we found that the concentration of *Alu* was considerably incremented in patients suffering SZ, and a significant difference was found between patients suffering from SZ and MDD or AIPD. Moreover, ROC analysis also showed that *Alu* was helpful in assist in the complementary diagnosis of SZ, and differentially diagnose between the patients with MDD or AIPD and patients with SZ. Furthermore, we also found a positive correlation between the *Alu* and IL-1β concentrations in patients with SZ, MDD, and AIPD, and between the concentrations of *Alu* and IL-18 in patients with SZ.

There are numerous kinds of psychiatric disorders, with diverse and overlapping clinical manifestations. It is difficult to distinguish the negative symptoms of SZ from MDD. Although the SZ and AIPD have different etiologies, they include some similar symptomatic and functional impairments. This causes challenges in diagnosing psychiatric disorders. The psychiatric disorders include some insignificant symptoms in the initial stages that are easily overlooked, however, it might represent a potential danger to individuals, families, and society. Hence, it is helpful to investigate the expression pattern and role of innovative molecular markers in psychiatric disorders.

*Alu* has been extensively utilized as a molecular biomarker in diagnosing various diseases (41, 42). Only a few studies exist on *Alu* in psychiatric disorders (43), moreover, the concentration of *Alu* was not investigated in patients and healthy controls. In addition, the correlations between the concentration of *Alu* and clinical characteristics were not studied. In the present study, we found that the *Alu* concentrations were incremented considerably in patients suffering from SZ, in comparison to healthy people. It was also found that the concentrations of *Alu* of patients with MDD and AIPD are significantly lower compared to the patients with SZ. In addition, ROC analysis showed the AUC 0.6732 between SZ patients and healthy individuals with a sensitivity of 51.83% and a specificity of 90%. Moreover, the AUC was 0.6690 between SZ patients and MDD patients with a 60.42% sensitivity and a 70.73% specificity, and the AUC was 0.6684 between SZ patients and AIPD patients with a 75.86% sensitivity and a 57.93% specificity. These findings show that *Alu* may be useful in the diagnosis of SZ, and may be a marker that distinguishes between SZ, MDD, and AIPD. However, the values of AUC are not yet ideal, and so the sensitivity and specificity of this potential biomarker would need further improvement. The progression and development of psychiatric disorders are caused by the interaction between genetic and environmental factors. Identifying diseases based on a single molecule is difficult. Future studies will increase the size and further characterize the patient group, and combine *Alu* with other indexes to enhance the sensitivity and accuracy of psychiatric disorders diagnosis.

Based on the analysis between *Alu* and clinical characteristics in patients with SZ, MDD, and AIPD, it was showed that no statistically significant difference exists in *Alu* in these various groups in terms of gender, current age, psychosis onset age, duration of psychosis, current treatment, family history, and duration of pharmacological treatment. There was no significant difference between *Alu* and CGI scores, abandon score, and attack score of patients with SZ. It indicated that *Alu* might be an independent indicator in relation to these indexes. *Alu* is released from cells, and the changes in the concentrations of *Alu* only represent changes at the cellular level. In addition to the variations in nerve cells, the clinical manifestations of patients with psychiatric disorders are also influenced by many other factors, including physique, character, and living environment of patients. This might be one of the reasons for the lack of a correlation between *Alu* and these indexes.

**Figure 3:**
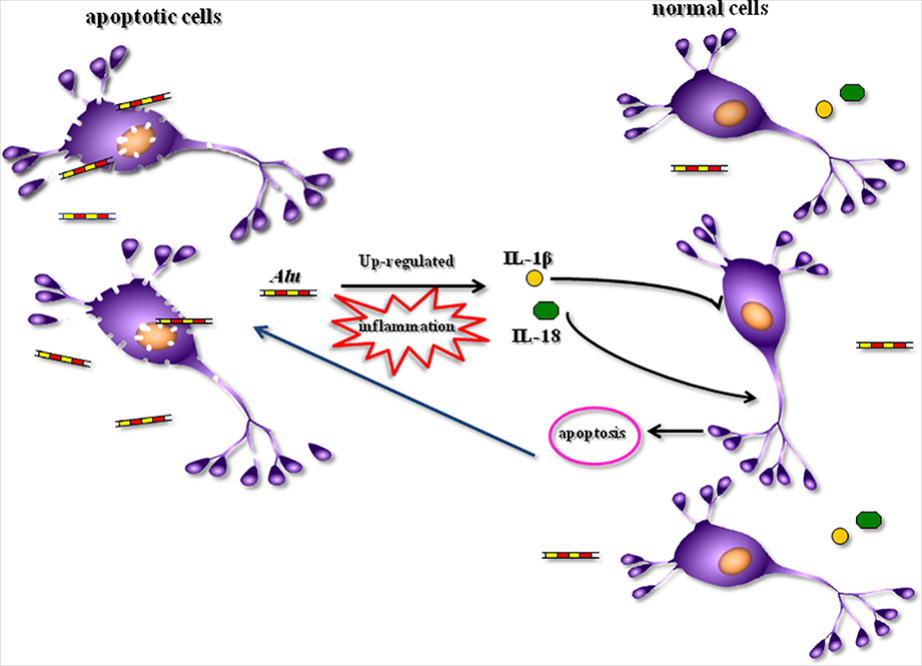
A hypothesis of the role of *Alu* sequences in the progression of psychiatric disorders. IL-1β, interleukin-1β; IL-18, interleukin-18.

The apoptosis of neuronal takes place within developing psychiatric disorders. Large quantities of cellular contents are released by apoptotic cells into the interstitial space of cells while triggering the inflammatory reactions (44, 45). Neuroinflammation can further enhance the progression of psychiatric disorders. Some inflammatory cytokines, including interleukin 10 (IL-10) (46), IL-1β, IL-6 (47), and interferon (IFN)-λ, are considerably incremented in patients with SZ. These indicators are related to the worse functional outcomes of patients with SZ (48). High levels of inflammation markers are linked to the vulnerability to anxiety disorders (49). A therapeutic impact was imposed by anti-inflammatory drugs on patients with psychiatric disorders (50). In recent years, it has been reported that *Alu* played a role also in the process of inflammation (51, 52). The injection of ApoExos containing unedited *Alu* repeats resulted in inflammation in mice (26). *Alu* had an influence on the inflammatory response mechanisms of atherosclerosis and coronary artery disease (53). The formation of NLRP3 inflammasome and upregulation of IL-18 and IL-1β subsequently were activated by the accumulation of *Alu* transcripts (36, 37). This process might have a role in both neuroinflammation and neurodegeneration related to Alzheimer’s disease (AD) and other degenerative brain disorders (54). Hence, in the present work, the correlation of *Alu* and inflammatory factor IL-18 and IL-1β was investigated. We found a positive correlation between *Alu* and IL-1β in patients with SZ, MDD, and AIPD. We also found a positive correlation between *Alu* and IL-18 in patients with SZ. Kim et al. found that the accumulation of *Alu* RNA led to the elaboration of IL-18 in human eyes with geographic atrophy (36), consequently, the activation of NLRP3 inflammasome and the apoptosis in an IL-18-dependent mode were triggered (55, 56). It was also found that *Alu* up-regulated the expression level of IL-1β within the endothelial dysfunction process (37). According to these contents, we propose a hypothesis that apoptosis occurred in progressing the psychiatric disorders while releasing large quantities of *Alu* sequences from apoptotic cells. Later, the axis of *Alu*-IL-18/IL-1β was activated and inflammation was triggered aggravating the progression and development of psychiatric disorders, in turn (**Figure 3**). As the future work, we will focus on the mechanism of psychiatric disorders based on this hypothesis.

In conclusion, the present study identified a considerable difference in *Alu*, between SZ patients and MDD, AIPD patients, and healthy individuals. Additionally, we also found a positive relation between IL-1β and the concentrations of *Alu* in patients with SZ, MDD, and AIPD, and between the concentrations of *Alu* and IL-18 in patients with SZ. It may indicate the *Alu* concentration might be helpful in the complementary diagnosis of psychiatric disorders, and *Alu*-IL-18/IL-1β might be involved in progressing the psychiatric disorders.

## Data Availability Statement

The datasets generated for this study are available on request to the corresponding author.

## Ethics Statement

The studies involving human participants were reviewed and approved by the Human Research Ethics Committee of the Affiliated Hospital of Nantong University. Written informed consent to participate in this study was provided by the participants’ legal guardian/next of kin.

## Author Contributions

JQ analyzed the data and wrote the paper, L-YC performed experiments. X-JS collected the samples. S-QJ designed the study.

## Funding

This work was supported by grants from The Six Talent Peaks Project of Jiangsu Province of China (2012-WS-119) and The Health and Planning Project of Nantong (QB201910).

## Conflict of Interest

The authors declare that the research was conducted in the absence of any commercial or financial relationships that could be construed as a potential conflict of interest.
